# Genome-Wide Identification of the *Dendrocalamus latiflorus* IDD Gene Family and Its Functional Role in Bamboo Shoot Development

**DOI:** 10.3390/genes16091036

**Published:** 2025-08-30

**Authors:** Yu-Han Lin, Peng-Kai Zhu, Mei-Yin Zeng, Xin-Ru Gao, Tian-You He, Jun-Dong Rong, Yu-Shan Zheng, Ling-Yan Chen

**Affiliations:** 1 College of Landscape Architecture and Art, Fujian Agriculture and Forestry University, Fuzhou 350002, China; kidult77@fafu.edu.cn (Y.-H.L.);; 2 College of Forestry, Fujian Agriculture and Forestry University, Fuzhou 350002, China

**Keywords:** gene family, IDD transcription factors, bamboo shoot development, *Dendrocalamus latiflorus*

## Abstract

**Background**: Transcription factors (TFs) critically regulate gene expression, orchestrating plant growth, development, and stress responses. The conserved IDD (*INDETERMINATE DOMAIN*) TF family modulates key developmental processes, including root, stem, and seed morphogenesis. *Dendrocalamus latiflorus* Munro, an economically vital sympodial bamboo in southern China, suffers significant yield losses due to prevalent bamboo shoot abortion, impacting both edible shoot production and timber output. Despite the documented roles of IDD TFs in shoot apical meristem expression and lateral organ regulation, their genome-wide characterization in *D. latiflorus* remains unstudied. **Methods**: Using IDD members from *Arabidopsis thaliana*, *Oryza sativa*, and *Phyllostachys edulis* as references, we identified 45 *DlIDD* genes in *D. latiflorus*. Comprehensive bioinformatics analyses included gene characterization, protein physicochemical assessment, phylogenetic reconstruction, and examination of gene structures/conserved domains. Differential expression of *DlIDD* genes was profiled between dormant and sprouting bamboo shoots to infer putative functions. **Results**: The 45 *DlIDD* genes were phylogenetically classified into three subfamilies and unevenly distributed across 34 chromosomes. Whole-genome duplication (WGD) events drove the expansion of this gene family. Promoter analyses revealed enriched cis-regulatory elements associated with hormone response and developmental regulation. Functional analyses suggested potential roles for *DlIDD* genes in bamboo shoot development. **Conclusions**: This study provides a foundation for future research to elucidate the functions of IDD TFs and their regulatory mechanisms in bamboo shoot morphogenesis and lateral bud development within woody monocots.

## 1. Introduction

Plant transcription factors orchestrate development by binding cis-elements to regulate gene transcription [1]. The IDD family, a plant-specific clade within the C_2_H_2_ zinc finger TF superfamily, is evolutionarily conserved across land plants [2]. Its name derives from maize *INDETERMINATE1* (ID1), the first characterized member [3]. IDD proteins are hybrid TFs containing dual C_2_H_2_ zinc fingers, two C_2_HC-type zinc fingers (with the N-terminal adopting a TFIIIA-type configuration), a nuclear localization signal, and flanking sequences, collectively forming the IDD [4].

Functionally, IDD TFs regulate diverse processes via protein interaction networks, governing cell/tissue fate, hormone signaling, and development across organs and stages. They play pivotal roles in seed germination [5,6,7,8], plant architecture and starch sheath development [9,10,11], hormone signaling [12], leaf/floral morphogenesis [13], and ammonium/phosphate homeostasis [14]. Studies in *A. thaliana* and *O. sativa* have experimentally validated the roles of numerous IDD genes, including the modulation of seed germination (*AtIDD1*), root development (*AtIDD3*, *AtIDD10*), and auxin-mediated organogenesis (*AtIDD14/15/16*) [15,16,17,18,19].

*D. latiflorus*, a large tropical sympodial bamboo native to southern China, is a vital multipurpose resource. Its high-value timber is used in construction and crafts, fibers in weaving/paper, foliage as food/tea, and tender shoots as a significant nutritional and economic commodity in Asia [20]. Propagation occurs primarily via asexual rhizome-borne axillary buds differentiating into new shoots, making bud development critical for yield. While studies in model bamboo species like *Phyllostachys edulis* (*P. edulis*) have provided foundational insights, the functional relevance of these findings across the diverse bamboo lineage requires careful examination. For instance, in P. edulis, *PheIDD21* regulates shoot bud development, showing sucrose-inducible and hormone-responsive expression, suggesting a master regulatory role in bud morphogenesis [21]. However, *D. latiflorus* possesses distinct biological and genomic characteristics that warrant a separate, dedicated analysis. As a sympodial (clumping) bamboo, its rhizome and shoot development patterns differ fundamentally from the monopodial (running) growth habit of *P. edulis.* Furthermore, *D. latiflorus* is a hexaploid with a larger and more complex genome, which likely has led to the expansion and functional diversification of gene families, including the IDD TFs. Therefore, extrapolating mechanisms directly from *P. edulis* may not fully capture the unique regulatory networks in *D. latiflorus*, particularly those governing its highly valued shoot development. A systematic study of the IDD family in *D. latiflorus* is thus crucial for elucidating species-specific mechanisms that control its agronomically critical traits.

Our laboratory’s 2022 chromosome-level assembly of the hexaploid *D. latiflorus* genome enabled this genome-wide study. We identified 45 candidate *DlIDD* genes. Comprehensive characterization included phylogenetic relationships, gene structures, conserved motifs, chromosomal distribution, duplication events, collinearity, cis-regulatory elements, and tissue-specific expression. Expression dynamics across key bamboo shoot developmental stages were quantified. This integrated analysis provides a foundational resource for understanding *DlIDD* gene functions potentially regulating shoot growth and development in *D. latiflorus*.

## 2. Materials and Methods

### 2.1. Genome-Wide Identification of DlIDD Family Members

To systematically identify IDD transcription factor family members in *D. latiflorus*, we initiated our analysis using the published genome assembly (Raw data from this study have been deposited in the NCBI Sequence Read Archive (SRA) under BioProject PRJNA600661. The assembly genome is available at NCBI under accession number JACBGG000000000 and forestry.fafu.edu.cn/pub/Dla). Reference protein sequences comprising 16 *AtIDD* genes from *A. thaliana* and 15 *OsIDD* genes from *O. sativa* were retrieved from NCBI (https://www.ncbi.nlm.nih.gov), followed by local BLASTp alignments against the *D. latiflorus* proteome with a stringent E-value threshold of 1 × 10^−10^ to identify preliminary candidates. Subsequent domain validation was performed through integrated interrogation of NCBI CDD, InterProScan (http://www.ebi.ac.uk/Tools/pfa/iprscan/, accessed on 2 April 2025), and SMART (http://smart.embl-heidelberg.de, accessed on 2 April 2025) databases, retaining only sequences harboring complete INDETERMINATE domains. Validated proteins underwent multiple sequence alignment via Clustal W [22] and phylogenetic reconstruction using the neighbor-joining method in MEGA11 with 1000 bootstrap replicates to resolve evolutionary relationships and subfamily classifications [23]. Finally, comprehensive biophysical characterization—including molecular weight (MW), isoelectric point (pI), instability index, and grand average of hydropathy (GRAVY)—was conducted using TBtools v2.326 “Protein Parameter Calc” module [24], with comparative analyses against orthologous proteins from reference species providing structural and functional insights.

### 2.2. Phylogenetic Reconstruction and Sequence Analyses Across Four Plant Species

Protein sequences for *A. thaliana*, *O. sativa*, and *P. edulis* were retrieved from TAIR (https://www.arabidopsis.org, accessed on 4 April 2025), Rice Data Center (https://www.ricedata.cn/gene/, accessed on 4 April 2025), and the Bamboo Genome Database (http://forestry.fafu.edu.cn/db/PhePacBio/phe/Jbnest.php, accessed on 4 April 2025, respectively.

To resolve evolutionary relationships among the four species, multiple sequence alignment of 16 *AtIDD*, 15 *OsIDD*, 31 *PheIDD*, and 45 *DlIDD* proteins was performed using ClustalW (https://www.genome.jp/tools-bin/clustalw, accessed on 4 April 2025) under default parameters [22]. The aligned sequences were subjected to phylogenetic reconstruction in MEGA 7.0 [25]. This version was specifically used for this cross-species phylogenetic analysis to ensure direct comparability with a large body of previously published work in plant comparative genomics that utilized MEGA7. For all other phylogenetic analyses within *D. latiflorus* (Section 2.1), the updated MEGA11 [23] was employed. The analysis was performed with complete deletion of missing data and 1000 bootstrap replicates to assess branch robustness.

For conserved domain analysis, the TCP domains (55–60 amino acid segments) of *DlIDD* proteins were extracted and aligned using DNAMAN 9.0 [26] to evaluate sequence conservation and diversification patterns. To investigate potential post-transcriptional regulation, miRNA-binding sites within *DlIDD* genes were predicted using RNA22 v2 (https://cm.jefferson.edu/rna22/Interactive/, accessed on 10 April 2025) [27] with default thresholds: hybridization energy ≤ −15 kcal/mol and seed region complementarity ≥ 7 nt.

### 2.3. Conserved Motif and Gene Structure Analysis

Gene structure analysis was performed using the *D. latiflorus* genome annotation file (GFF3 format; available at http://forestry.fafu.edu.cn/pub/Dla/Download_Genome.html, accessed on 25 April 2025) and coding sequences (CDSs). Exon–intron organization of *DlIDD* genes was resolved through TBtools v2.326 [24], with structural features visualized via its integrated graphics module. Concurrently, conserved protein motifs were systematically identified using MEME 5.4.1 (http://meme-suite.org/tools/meme, accessed on 25 April 2025) under the following parameters: maximum motifs = 15, and motif width = 6–50 amino acids, with other settings at default. Further characterization included transmembrane domain (TMD) prediction via TMHMM Server 2.0 (http://www.cbs.dtu.dk/services/TMHMM/, accessed on 25 April 2025) [28] and nuclear localization signal (NLS) analysis using cNLS Mapper (https://nls-mapper.iab.keio.ac.jp/, accessed on 25 April 2025), collectively informing subcellular localization patterns. All analytical results were integrated and visualized as comprehensive gene architecture and functional domain maps using TBtools v2.326.

### 2.4. Chromosomal Mapping, Synteny, and Gene Duplication Analysis

Chromosomal localization and duplication events of *DlIDD* genes were analyzed using the *D. latiflorus* genome annotation. MCScanX (default parameters) classified duplication events into tandem and segmental duplications based on genomic positional relationships. Chromosomal distribution and syntenic relationships were visualized via Circos v0.69, with syntenic gene pairs indicated by arched connectors. To elucidate evolutionary patterns, cross-species synteny analysis of IDD proteins among *D. latiflorus*, *A. thaliana*, *O. sativa*, and *P. edulis* was performed using the “Dual Synteny Plot” module in TBtools v2.326. Concurrently, selection pressures acting on duplicated gene pairs were assessed through Ka/Ks calculations [29] using the Ka/Ks Calculator, where Ka/Ks ratios >1, =1, and <1 indicated positive selection, neutral evolution, and purifying selection, respectively.

### 2.5. Promoter Cis-Acting Element Analysis

To characterize regulatory elements within *DlIDD* gene promoters, we extracted 2000 bp upstream sequences of the translation start site (ATG) from the *D. latiflorus* genome using TBtools v2.326. These promoter sequences were subjected to cis-acting element prediction via the PlantCARE database (http://bioinformatics.psb.ugent.be/webtools/plantcare/html/, accessed on 13 May 2025) [30], with specific focus on elements associated with hormone response (e.g., ABRE), stress adaptation (e.g., MYB-binding sites), and developmental regulation (e.g., G-box). Predicted elements’ distribution and functional associations were subsequently visualized through the Gene Structure Display Server (GSDS 2.0; http://gsds.gao-lab.org/, accessed on 13 May 2025), generating comprehensive promoter architecture maps.

### 2.6. Protein–Protein Interaction (PPI) Network Construction

Putative interaction networks of *DlIDD* proteins were predicted through cross-species orthology mapping using the STRING v11.0 database (http://string-db.org/, accessed on 2 June 2025) [31], leveraging the comprehensively annotated *A. thaliana* PPI network as a reference. Orthologous sequences between *A. thaliana* and *D. latiflorus* IDD proteins were identified via BLASTP alignments with a stringent E-value cutoff ≤ 1 × 10^−5^. High-confidence interactions (confidence score ≥ 0.7) were retained to construct the core *DlIDD* PPI network. The resulting network was visualized in Cytoscape v3.9.1 [32], employing a Force-Directed layout algorithm to reveal topological relationships. Hub genes were annotated based on node degree and modular clustering analysis using the MCODE plugin, with node color and size mapped to functional classifications and interaction connectivity.

### 2.7. Plant Materials and Growth Conditions

*D. latiflorus* specimens cultivated at Fujian Agriculture and Forestry University, China (26°05′ N, 119°18′ E), served as experimental material. Bamboo shoot samples were collected during distinct developmental stages in May 2024: the dormant phase (physiological quiescence) and sprouting phase (from bud swelling to soil emergence). Plants were maintained under controlled conditions: 12 h photoperiod, constant temperature (25 ± 2 °C), and relative humidity (70% ± 3%). Three independent biological replicates were established per developmental stage to mitigate biological variability, each comprising root tissues pooled from three randomly selected healthy plants. Collected samples underwent surface sterilization with 75% ethanol (30 s), triple-rinsing in sterile water, flash-freezing in liquid nitrogen, and subsequent storage at −80 °C until molecular analysis.

### 2.8. Transcriptome Sequencing and Expression Profiling of DlIDD Genes

Transcriptome sequencing of *D. latiflorus* samples was performed using the Illumina HiSeq X Ten platform. Raw sequencing data underwent quality control via Trimmomatic v0.39 [33], implementing adapter removal, low-quality base trimming (Phred score < Q20), and elimination of reads < 50 bp. High-quality clean reads were aligned to the *D. latiflorus* reference genome using HISAT2 v2.2.1 under default parameters, with uniquely mapped reads retained for downstream analysis. Gene expression levels were quantified as transcripts per million (TPM) values using TPMCalculator [34], considering genes with mean TPM ≥ 0.1 as expressed.

Differential expression analysis was subsequently conducted using the DESeq2 module integrated into TBtools v2.326 [24]. Significantly differentially expressed genes (DEGs) were identified using stringent thresholds: |log_2_(fold change)| ≥ 1.5 and Benjamini–Hochberg adjusted *p*-value (padj) < 0.05. Pairwise comparisons between developmental stages (e.g., dormant vs. sprouting) revealed stage-associated DEGs, with expression patterns visualized through hierarchical clustering heatmaps.

### 2.9. Quantitative Real-Time PCR (qRT-PCR) Validation

Quantitative real-time PCR (qRT-PCR) was employed to validate expression patterns of selected genes during dormant and sprouting bamboo shoot stages, utilizing three biological replicates per experimental group. Gene-specific primers were designed via the NCBI Primer-BLAST online tool (https://www.ncbi.nlm.nih.gov/tools/primer-blast/, accessed on 28 August 2025), with sequences detailed in Table S5. Total RNA extraction was performed using the FastPure^®^ Universal Plant Total RNA Isolation Kit RC411 (Vazyme, Nanjing, China), followed by cDNA synthesis with Hifair^®^ AdvanceFast One-step RT-gDNA Digestion SuperMix for qPCR (Yeasen, Shanghai, China).

qRT-PCR amplification reactions were conducted on an Applied Biosystems QuantStudio 3 Real-Time PCR System (Thermo Fisher Scientific, Waltham, MA, USA) using Hieff^®^ qPCR SYBR Green Master Mix (Low Rox Plus; Yeasen, Shanghai, China). The thermal cycling protocol comprised an initial denaturation at 95 °C for 5 min, followed by 40 cycles of denaturation at 95 °C for 10 s and combined annealing/extension at 60 °C for 30 s, with dissociation curve analysis performed through a temperature gradient from 60 °C to 95 °C (0.3 °C increments per 5 s hold).

Relative gene expression levels were calculated using the 2^−ΔΔCt^ method with glyceraldehyde-3-phosphate dehydrogenase (GAPDH) as the endogenous reference gene, and differential expression was quantified as fold-change values.

## 3. Results

### 3.1. Identification of DlIDD Genes and Physicochemical Characterization of Encoded Proteins

Using *A. thaliana* and *O. sativa* IDD family members as references, we identified 45 *DlIDD* genes in the *D. latiflorus* genome via conserved domain analysis and gene structure screening. These genes were systematically designated as *DlIDD1–14* (subgenome A), *DlIDD15–33* (subgenome B), and *DlIDD34–45* (subgenome C) based on their subgenomic origins and chromosomal localization. Phylogenetic classification revealed that the majority of *DlIDD* genes belong to the PCF subfamily (35 members), with the CIN subfamily (21 members) and CYC/TB1 subfamily (10 members) comprising the remainder, mirroring distribution patterns observed in *A. thaliana*, *O. sativa*, and *P. edulis*.

Further analysis demonstrated substantial diversity in *DlIDD* protein properties: molecular weights ranged from 16.24 kDa (*DlIDD45*) to 71.78 kDa (*DlIDD17*), amino acid lengths varied between 148 and 668 residues, isoelectric points spanned 5.23 to 9.57, instability indices ranged from 39.30 to 77.28, and grand average of hydropathy (GRAVY) values varied between −0.632 and −0.307. Subcellular localization predictions indicated nuclear predominance for most *DlIDD* proteins, consistent with their functional roles as transcription factors (Table S1).

### 3.2. Phylogenetic Analysis of IDD Gene Families Across Four Plant Species

**Figure 1:**
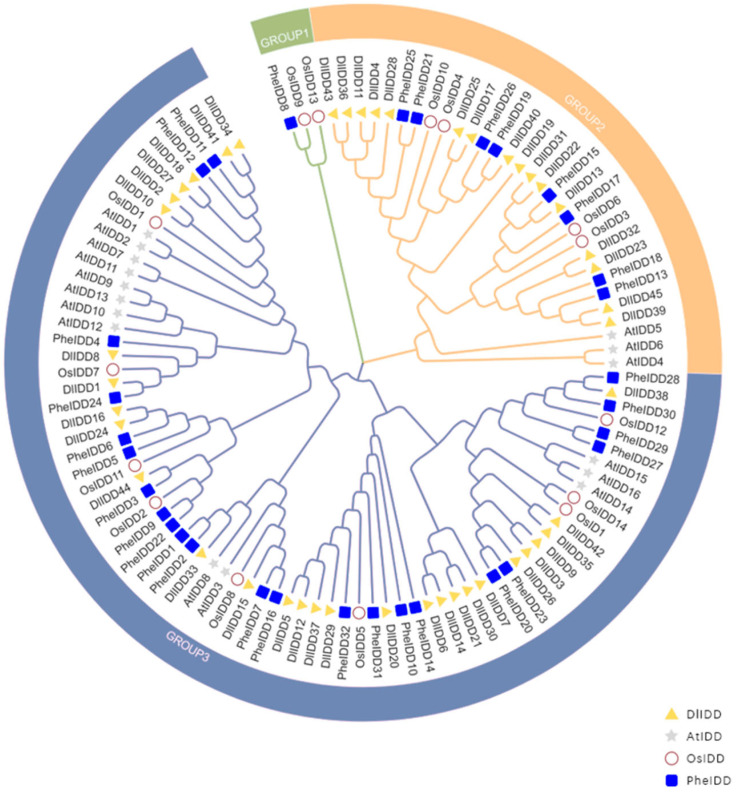
Neighbor-joining phylogenetic tree of IDD transcription factors across *A. thaliana*, *O. sativa*, *D. latiflorus*, and *P. edulis*. The different-colored branches represent distinct subclades: clade I (green), clade II (orange), and clade III (blue). Four different symbols represent four species.

To elucidate evolutionary relationships among IDD genes, we aligned full-length protein sequences from *D. latiflorus* (45 *DlIDD*), *O. sativa* (15 *OsIDD*), *P. edulis* (32 *PheIDD*), and *A. thaliana* (16 *AtIDD*), constructing a phylogenetic tree comprising 108 orthologs (Figure 1). Phylogenetic reconstruction grouped all IDD proteins into three distinct clades (G1–G3), where clade G1 exclusively comprised two *O. sativa* (*OsIDD13*, *OsIDD9*) orthologs and one *P. edulis* (*PheIDD8*) ortholog, suggesting lineage-specific conservation in monocots.

Comparative analysis revealed asymmetric subfamily distributions between *D. latiflorus* and *A. thaliana*, with PCF predominating in both lineages. This conservation pattern suggests IDD gene family diversification preceded the monocot–eudicot divergence. The topology revealed pronounced sequence conservation between bamboo species, manifested by *DlIDD28* and *PheIDD38* forming a cluster, along with *DlIDD1* and *PheIDD24* within conserved clades, confirming close orthologous relationships among bambusoid taxa.

Notably, *D. latiflorus* displayed substantial gene family expansion, containing 3-fold more IDD genes than *O. sativa* (15 members) and 1.4-fold more than *P. edulis* (32 members). Neighbor-joining analysis further showed clustered distributions of *DlIDD* genes across phylogenetic branches, implicating polyploidization as the primary expansion mechanism (Figure 1).

### 3.3. Gene Structure and Conserved Protein Motif Analysis of DlIDD Genes

To systematically characterize structural features and functional conservation within the *D. latiflorus* IDD gene family, we integrated exon–intron architecture and protein motif information across all 45 *DlIDD* members.

**Figure 2:**
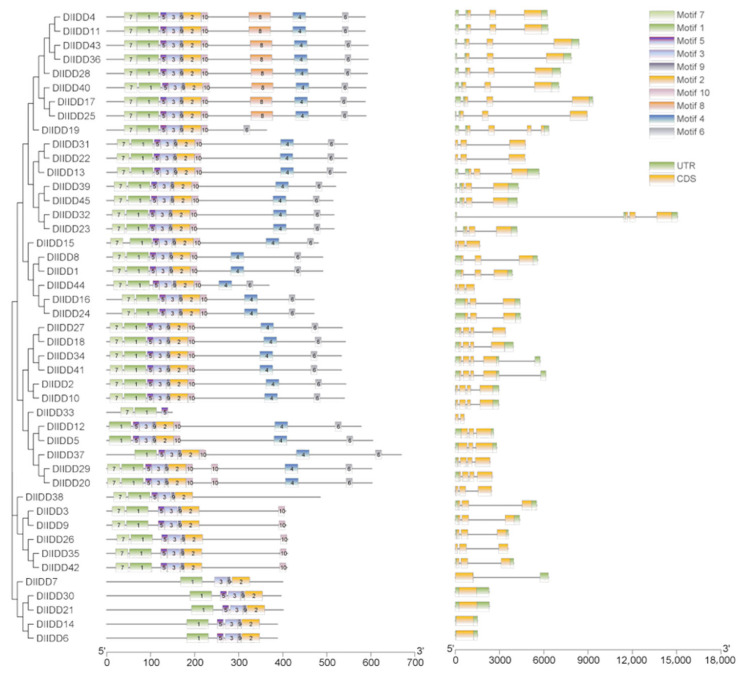
Gene structure architecture and conserved motif organization of *DlIDD* family members in *D. latiflorus.* The left panel shows the phylogenetic tree of 45 DlIDD proteins. The middle panel presents the distribution of conserved motifs (Motifs 1–10) identified by MEME, with different colors indicating distinct motifs. The right panel illustrates the exon–intron organization of DlIDD genes, where orange boxes represent coding sequences (CDSs), green boxes represent untranslated regions (UTRs), and black lines indicate introns.

Exon number analysis revealed substantial variation (1–4 exons per gene), with the majority (e.g., *DlIDD9*, *DlIDD40*, *DlIDD42*) containing 3 exons. In contrast, five genes (*DlIDD6*, *DlIDD14*, *DlIDD21*) possessed a single exon (Figure 2). Notably, several genes (*DlIDD10*, *DlIDD33*) lacked 5′-UTRs, while five members (*DlIDD22*, *DlIDD31*) lacked both 5′ and 3′ UTRs, suggesting potential noncanonical transcriptional regulatory mechanisms. Among multi-exon genes (≥2 exons), most displayed elongated terminal exons at the 3′ end (e.g., *DlIDD28*, *DlIDD36*), with rare exceptions such as *DlIDD19*, reflecting evolutionary diversity potentially driven by gene duplication or annotation artifacts.

Orthologous gene comparisons demonstrated conserved intron insertion positions and phases within phylogenetic clades (e.g., *DlIDD3* and its paralogs *DlIDD9* and *DlIDD26*), with identical intronic architectures among subfamily members, indicating strong purifying selection on core functional domains.

MEME analysis identified 14 conserved motifs (Motifs 1–14) in *DlIDD* proteins, wherein Motif 2 (C_2_H_2_ zinc finger) and Motif 4 (C_2_HC zinc finger) constituted the core ID-domain modules (Figure 2). Whereas N-terminal motifs (Motifs 1–5) exhibited strong conservation, C-terminal motifs (Motifs 6–14) displayed significant structural divergence, potentially underlying functional specialization and species-specific regulation. For instance, Motif 7 (TRDFLG) and Motif 8 (MSATALLQKAA) were predicted to mediate protein–protein interactions, whereas Motif 12 (ELQLLP) likely functions as a transcriptional repressor domain, analogous to the Arabidopsis EAR motif.

### 3.4. Chromosomal Distribution and Gene Duplication Events of DlIDD Genes

**Figure 3:**
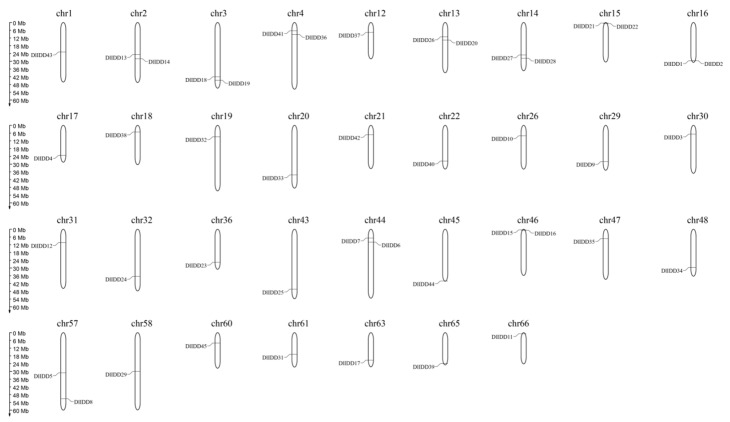
Chromosomal distribution landscape of *DlIDD* gene family members in *D. latiflorus*, revealing subtelomeric clustering bias.

Chromosomal mapping using MG2C revealed that the 45 *DlIDD* genes are distributed across 34 chromosomes in *D. latiflorus*, displaying significant spatial bias: 78% (35 genes) localize to subtelomeric regions, whereas only 22% (e.g., *DlIDD29*) occupy interstitial positions (Figure 3). Notably, *DlIDD18* and *DlIDD19* form a tandem duplication array at the terminus of chromosome 3p. In contrast, *DlIDD5* and *DlIDD8* are positioned in mid-arm and q-terminus regions of chromosome 57, suggesting that subtelomeric domains may represent evolutionary hotspots for IDD gene diversification.

**Figure 4:**
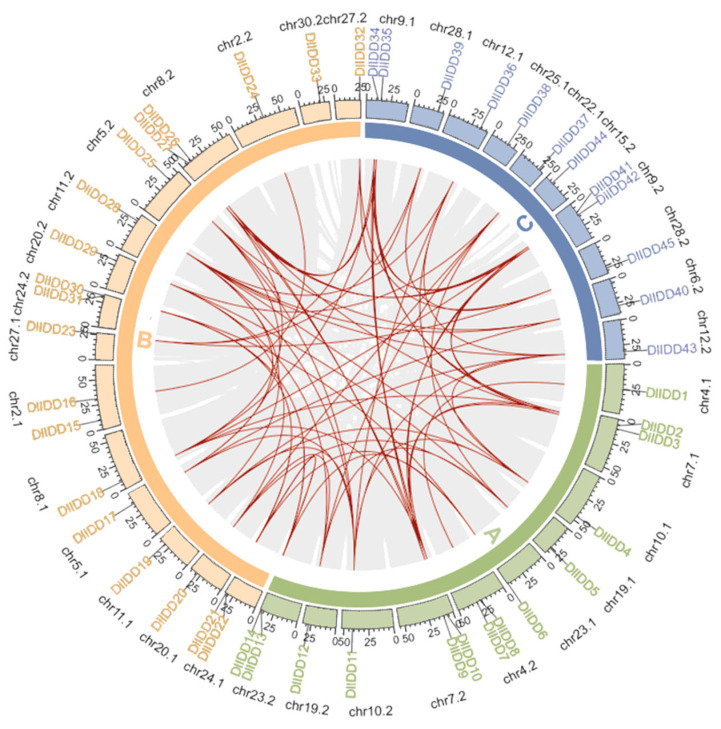
Chromosomal distribution and synteny analysis of *DlIDD* gene family members in *D. latiflorus*. The outermost circle represents the 27 chromosomes of *D. latiflorus*, with different colors (A–C) indicating distinct chromosomal groups. Gene IDs labeled around the circle correspond to the physical positions of *DlIDD* genes. The gray lines in the background indicate genome-wide syntenic blocks, while the red curves highlight collinear *DlIDD* gene pairs. This analysis suggests that whole-genome duplication events contributed to the expansion of the *DlIDD* gene family.

Syntenic analysis revealed complex collinearities among *DlIDD* members, identifying whole-genome duplication (WGD) as the predominant expansion mechanism (Figure 4). Pronounced subgenomic divergence was evident: chromosomes Chr29.1 (A), Chr30.1 (B), and Chr31.1 (C) lacked detectable IDD genes, while Chr18.1 (A) harbored six *DlIDD* genes (three PCF, two CIN, one CYC/TB1), indicating functional specialization across subgenomes. Tandem duplication events were comparatively rare, with a single validated pair (*DlIDD5-B*/*DlIDD6-B*) potentially enabling neofunctionalization via localized gene duplication.

### 3.5. Synteny and Positive Selection Analysis of IDD Gene Families

**Figure 5:**
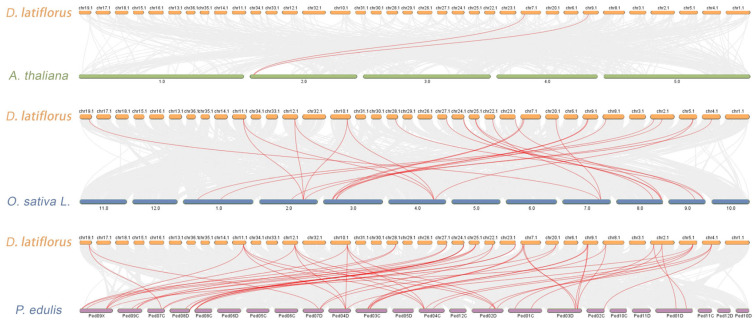
Comparative synteny analysis of IDD gene families among *D. latiflorus*, *A. thaliana*, *O. sativa*, and *P. edulis*. The bars represent the chromosomes (or scaffolds) of each species, with *D. latiflorus* shown at the top of each comparison. Red curves highlight syntenic IDD gene pairs between *D. latiflorus* and the corresponding species, while gray lines in the background denote genome-wide collinear blocks. This analysis reveals both conserved and species-specific synteny relationships, reflecting the evolutionary divergence of IDD genes across monocots and dicots.

Across Species McScanX-based synteny analysis revealed exceptionally conserved evolutionary relationships between *D. latiflorus* and *P. edulis* (Figure 5), both belonging to Bambusoideae, with significantly more syntenic gene pairs than distantly related *A. thaliana*. Specifically, we identified 41 orthologous pairs between *D. latiflorus* and *P. edulis* (DL-PE), and 26 pairs with *O. sativa* (DL-OS), but only 2 homologous pairs with *A. thaliana* (DL-AT), corroborating the close phylogenetic affinity within Bambusoideae. Additionally, 14 paralogous pairs within *D. latiflorus* (DL-DL) reflected multiple rounds of whole-genome duplication (WGD) events in this hexaploid genome (Table S2).

The *DlIDD* gene family expansion (45 members) substantially exceeds diploid relatives (e.g., 15 in rice), aligning with its hexaploid architecture. Synteny and Ks analyses indicated that three paleopolyploidization events (ρ, σ, τ) likely facilitated gene retention through conserved duplication mechanisms. This expansion provides genetic substrates for subfunctionalization or neofunctionalization, enhancing ecological adaptability, exemplified by the CYC/TB1 subfamily expansion to 10 members, potentially enabling tiller bud regulation in dense bamboo habitats.

Selection pressures on duplicated genes were assessed via Ka/Ks ratios [35]. Most IDD gene pairs exhibited Ka/Ks < 1 (range: 0.06–0.93), with only one pair at 1.2, indicating predominant purifying selection to maintain core functions. Notably, *D. latiflorus* paralogs (DL-DL) showed a lower average Ka/Ks of 0.29, suggesting relaxed selection pressures conducive to functional diversification (Table S3).

### 3.6. Identification and Analysis of Cis-Regulatory Elements in DlIDD Gene Promoters

**Figure 6:**
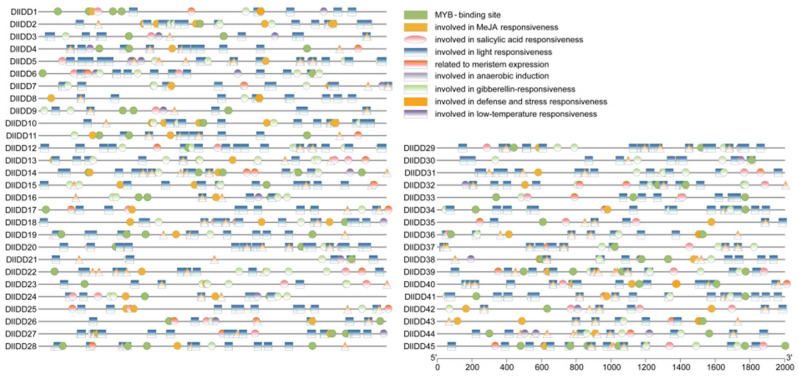
Cis-regulatory element profiling in *DlIDD* gene promoters reveals hormonal and environmental response networks. Element annotations: MYB-binding site; MeJA-responsive element; SA-responsive element; light-responsive element; meristem-specific regulatory element; anaerobic induction element; gibberellin-responsive element; defense/stress-responsive element; low-temperature responsive element.

We extracted 1500 bp promoter sequences upstream of the translation start site (ATG) for all 45 *DlIDD* genes to elucidate transcriptional regulation mechanisms. We predicted cis-regulatory elements using the PlantCARE database (Figure 6). Fourteen functional element categories were identified, primarily associated with hormonal responsiveness (e.g., ABA, auxin, gibberellin), developmental regulation (e.g., meristem specification, light response), and stress adaptation (e.g., low temperature, drought, defense response).

CYC/TB1 subfamily promoters exhibited significant enrichment of seed-specific regulatory elements (e.g., RY-repeats) [36], at two- to threefold higher frequencies than in the PCF and CIN subfamilies. For instance, *DlIDD12-C* (CYC/TB1) contained three RY-repeats, whereas *DlIDD7-A* (PCF) possessed only one. This implies specialized roles in bamboo shoot germination physiology or seed developmental programs. Additionally, low-temperature responsive elements (LTR) and MYC recognition sites (MBS) were enriched in stress-associated subfamilies (e.g., CIN), indicating potential modulation of environmental signaling for bamboo adaptability [37]. Further analysis revealed MYB-binding sites (MBSI, MBSII) in 81% of *DlIDD* promoters and WRKY-binding elements (W-box) in 65%. *DlIDD12-C* contained five MYB sites and one WRKY site, potentially enabling integrated transcriptional regulation through multifactorial signaling cascades.

The diversity of cis-regulatory elements provides a molecular basis for functional divergence within the IDD family. ABA-responsive elements (ABREs) were enriched in *DlIDD34-B* (PCF), while jasmonate-responsive motifs (CGTCA) predominated in *DlIDD9-C* (CIN), suggesting subfamily-specific hormonal coordination during development and stress adaptation (Table S4).

### 3.7. Protein–Protein Interaction Network Construction and Functional Interpretation of DlIDD Proteins

**Figure 7:**
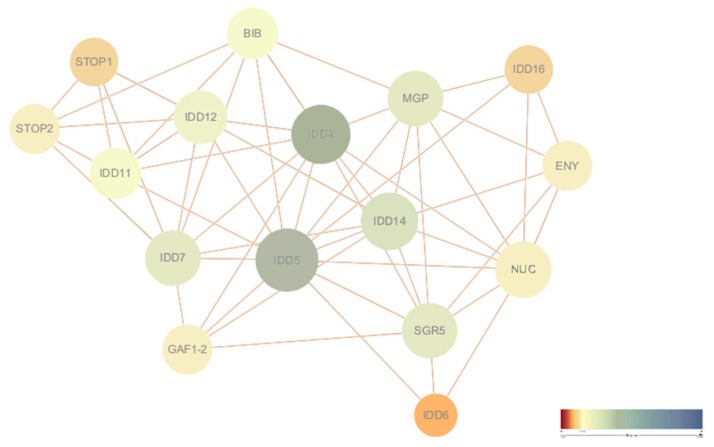
Predicted protein–protein interaction network of IDD proteins in *D. latiflorus.* Nodes represent *DlIDD* proteins or their predicted interacting partners, with node size proportional to the degree of connectivity (number of interactions). Edges indicate predicted protein–protein associations. The color gradient (from yellow to green to blue) reflects interaction confidence scores, with warmer colors representing lower confidence and cooler colors representing higher confidence.

To systematically decipher molecular regulatory mechanisms, we constructed a *DlIDD* protein interaction network (Figure 7) by submitting all 45 *DlIDD* sequences to the STRING database and performing orthology mapping against the Arabidopsis-annotated interactome. The resulting network revealed complex interconnections among *DlIDD* members organized into distinct functional modules.

Within the core interaction module, *DlIDD4* directly interacts with *DlIDD5*, with both proteins associating with meristem-activity regulators (e.g., MGP, SRG5) [38], suggesting their coordinated regulation of shoot apical meristem development through hormonal signaling integration [39]. A second interaction cluster comprised ENY (embryogenesis-associated proteins), NUC (nucleic acid-binding proteins), MJC (jasmonate signaling components), and IDD14 (Arabidopsis homolog), implying roles in cell fate determination and stress response during shoot development.

Notably, prior studies identified bamboo shoot development regulators functionally linked to IDD networks: *PheIDD* modulates branch bud initiation in Phyllostachys edulis [21], while opposing expression dynamics of PheMOC1a (downregulated) and PheMOC1b (upregulated) during shoot development reflect stage-specific regulation. Additionally, *PheIDD7* potentially mediates root nutrient absorption and metabolism, establishing direct/indirect functional connections within this network [21].

While predictive interactions provide valuable mechanistic insights, certain relationships (e.g., *DlIDD14*-SRG5) require experimental validation through yeast two-hybrid (Y2H) or bimolecular fluorescence complementation (BiFC) assays. Furthermore, bamboo-specific regulators (e.g., shoot-development miRNAs) remain underrepresented in existing databases, potentially limiting network comprehensiveness.

### 3.8. Expression Profiling of IDDs in Germinating Buds, Dormant Buds, and Distinct Organs

**Figure 8:**
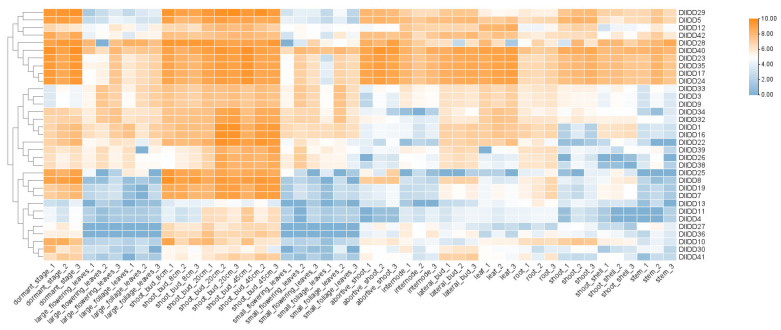
Heatmap depicting organ-specific expression patterns of IDD genes in *D. latiflorus.* The heatmap was drawn based on the log2 (TPM+1) values. TPM: transcripts per million mapped reads. The size and color scales represent expression levels from low to high.

The tissue-specific expression patterns of the IDD gene family provide critical insights into their functional roles during *D. latiflorus* development. Transcriptome-wide analysis revealed pronounced spatiotemporal expression heterogeneity among 45 *DlIDD* genes across six-week-old bamboo shoots, roots, stems, and leaves, as well as dormant and germinating shoot buds (Figure 8). The results demonstrate marked organ-specific expression divergence among IDD paralogs, with particularly pronounced transcript accumulation in shoots and leaves. For instance, *DlIDD5*, *DlIDD28*, and *DlIDD40* exhibited high transcript levels in nascent shoots, while *DlIDD24* was explicitly enriched in foliar tissues. *DlIDD40* demonstrated pronounced expression dominance in root systems, suggesting its possible involvement in the putative regulatory networks governing root morphogenesis.

**Figure 9:**
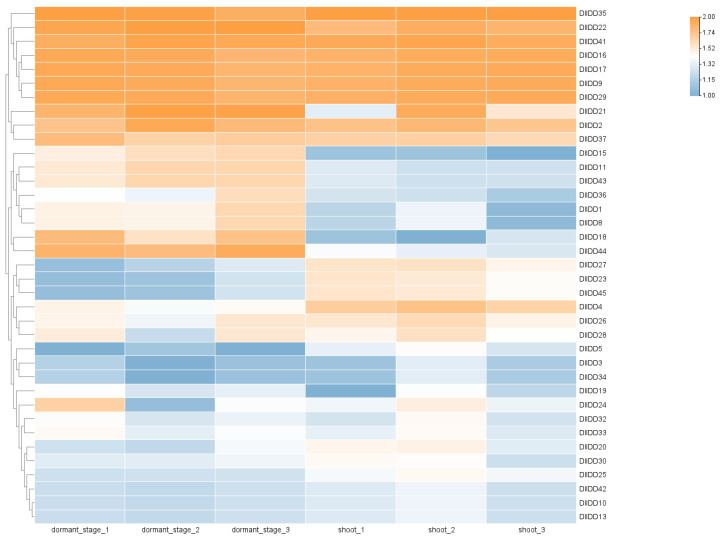
Heatmap visualization of differential expression patterns in *D. latiflorus* IDD genes across the dormancy-to-germination developmental continuum. The heatmap was drawn based on the log2 (TPM + 1) values. TPM: transcripts per million mapped reads. The size and color scales represent expression levels from low to high.

**Figure 10:**
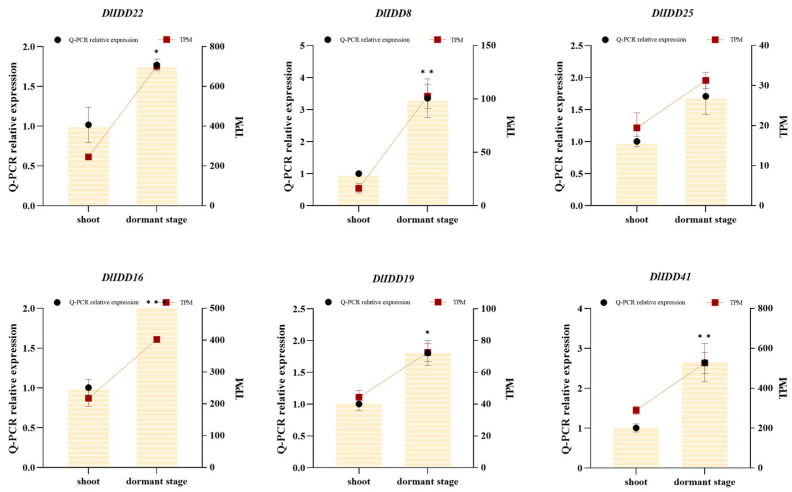
Temporal expression profiling of IDD genes in *D. latiflorus* shoots across developmental transitions. An asterisk indicates a significant difference in relative expression levels by qRT-PCR compared to the bamboo shoot stage. (* *p* < 0.05, ** *p* < 0.01, *** *p* < 0.001).

Further analysis of expression dynamics across shoot bud developmental stages identified 29 *DlIDD* genes as differentially expressed genes (DEGs), comprising 9 significantly upregulated and 20 downregulated members (Figure 9). Notably, *DlIDD44* and *DlIDD18*—belonging to the CYC/TB1 subfamily—exhibited substantially diminished transcript abundance during shoot germination. This expression attenuation aligns with the established molecular mechanism whereby TB1 orthologs regulate development by suppressing axillary bud outgrowth [40]. Furthermore, the cohort of downregulated genes progressively diminished while upregulated genes increased incrementally from dormancy to germination, indicating stage-specific temporal regulation by IDD members during bud morphogenesis. Specific genes (e.g., *DlIDD12* and *DlIDD34*) may participate in signaling initiation in early germination, whereas others (e.g., *DlIDD7* and *DlIDD29*) exhibit pronounced transcriptional activity during subsequent organogenesis, implying their potential role in this process. These expression dynamics were corroborated through qRT-PCR validation of six pivotal genes (*DlIDD5, DlIDD18, DlIDD24, DlIDD34, DlIDD40*, and *DlIDD44*), thereby confirming the reliability of transcriptomic profiling (Figure 10).

## 4. Discussion

IDD transcription factors represent a plant-specific subclass of zinc finger transcriptional regulators characterized by high evolutionary conservation, predominantly orchestrating plant developmental processes [41]. In this study, we identified 45 *DlIDD* family members through a local BLASTp search against the *D. latiflorus* proteome database, employing 16 *AtIDD*, 15 *OsIDD*, and 32 *PheIDD* reference sequences as queries [42]. The *DlIDD* genes were categorized into three major clades based on subfamily affiliation and chromosomal localization: subgenome A *(DlIDD1–14*; 14 members), subgenome B (*DlIDD15–33*; 19 members), and subgenome C (*DlIDD34–45*; 12 members). Domain integrity assessment of putative *DlIDD* genes via comparative alignment with rice IDD amino acid sequences revealed that—consistent with *A. thaliana*, *O. sativa*, and *P. edulis*—the majority of *DlIDDs* (35 members) belong to the PCF subfamily. CIN and CYC/TB1 subfamilies comprise 21 and 10 members, respectively. Cross-species comparative analysis indicates substantial structural and functional divergence among IDD transcription factors across plant lineages [43].

Phylogenetic reconstruction via multiple protein sequence alignment and neighbor-joining analysis elucidated the evolutionary conservation of IDDs. The resulting cladogram revealed pronounced structural homology between *D. latiflorus* and *P. edulis* IDD proteins, indicating their recent evolutionary divergence [44]. Cross-species comparative assessment demonstrated markedly asymmetric subfamily distribution between *D. latiflorus* and *A. thaliana* IDD genes, particularly evidenced by the near absence of CYC/TB1 subfamily representatives in *A. thaliana*. This phylogenetic pattern suggests ancestral IDD paralogs likely originated before the monocot–dicot divergence [45,46]. Notably, the IDD gene repertoire in *D. latiflorus* (45 members) substantially exceeds that of *A. thaliana* (16 members), with this expansion magnitude attributable to the hexaploid genome architecture and recurrent polyploidization events during Poaceae evolution [47]. Such divergence may reflect lineage-specific environmental selective pressures driving functional diversification through subfunctionalization or neofunctionalization of gene duplicates. For instance, the expanded CYC/TB1 subfamily in *D. latiflorus* may confer adaptive advantages in tillering regulation [48,49]. Furthermore, comparative genomics revealed a 3-fold expansion relative to *O. sativa* (15 members) and a 1.4-fold expansion versus *P. edulis* (32 members). This differential scaling likely stems from successive whole-genome duplication (WGD) events—including paleopolyploidization and lineage-specific polyploidy—that facilitated gene duplication and subsequent functional divergence, thereby potentiating adaptive evolution of the IDD family in *D. latiflorus* [50,51,52].

Whole-genome duplication (WGD) events constitute the primary driver of IDD gene family expansion in *D. latiflorus* [47]. Chromosomal localization analysis based on syntenic positional data revealed a non-uniform distribution of *DlIDD* members across the genome. At the same time, tandem duplication events can potentiate functional innovation and genomic diversification [53]. Only one tandem duplicate pair (*DlIDD-B* and *DlIDD6-B*) was identified, indicating that WGD-driven expansion coupled with functional conservation represents the dominant evolutionary mode, with tandem duplication serving merely as an auxiliary mechanism. Evolutionary constraint analysis of putative orthologs and ohnolog pairs demonstrated that all calculated Ka/Ks ratios were below 0.2726 (Ka/Ks < 1), consistent with the action of pervasive purifying selection during the family’s diversification [54]. Furthermore, cross-species comparison confirmed Ka/Ks < 1 for homologous gene pairs across four examined species, demonstrating strong purifying selection acting on *DlIDD* ortholog lineages.

Promoter cis-element profiling of IDD genes revealed that phylogenetically clustered members (e.g., *DlIDD21-A*, *DlIDD18-B*, *DlIDD24-C*) exhibit conserved architectures in motif composition and spatial distribution. Notably, *DlIDD21-A* and *DlIDD24-C* each harbor two light-responsive elements (G-boxes) and one gibberellin-responsive element (P-box), suggesting their coordinated regulation of photoperiod-dependent developmental processes [55,56]. Furthermore, CYC/TB1 subfamily promoters display enriched density of seed-specific regulatory motifs compared to other paralogs, pointing to their potential functional specialization during germination physiology [57,58,59].

The high economic value of *D. latiflorus* is compromised by prevalent shoot abortion phenomena, highlighting the importance of research into the molecular mechanisms governing shoot bud development. IDD transcription factors are established regulators of organogenesis and stage-specific development across diverse plant species [60]. Our tissue-specific expression profiling revealed pronounced transcriptional divergence among IDD paralogs, supporting the hypothesis of their specialized roles in orchestrating organ differentiation and morphogenesis in *D. latiflorus*. Temporal analysis of dormant-to-germinating bud transitions demonstrated progressive attenuation of downregulated genes concomitant with incremental upregulation, consistent with a model of stage-specific temporal regulation by IDD members during shoot development. However, future functional studies (e.g., through gene editing or transgenesis) are required to definitively establish the causal roles of specific DlIDD genes in these processes [56,57,58,59,60,61,62].

Collectively, these findings provide a valuable foundation for understanding the molecular underpinnings of IDD gene family functionality in rapid growth and architectural patterning in bamboo. Furthermore, they identify promising genetic targets for further functional validation, which could ultimately contribute to enhancing shoot yield and stress resilience through precision breeding strategies [63,64,65,66,67,68,69,70].

## 5. Conclusions

This study identified 45 IDD gene family members in *D. latiflorus*, conducting comparative genomic analyses against *A. thaliana*, *O. sativa*, and *P. edulis*. Phylogenetic reconstruction classified these genes into three evolutionarily distinct subfamilies. Structural characterization revealed conserved protein architectures and domain organization within each subfamily. Collinearity analysis demonstrated that whole-genome duplication events constitute the predominant driver of *DlIDD* family expansion, with tandem duplication representing a comparatively rare occurrence. Previous investigations have implicated numerous IDD orthologs in regulating shoot bud development. Leveraging transcriptomic datasets, we deciphered stage-specific expression dynamics of IDD genes across bamboo developmental transitions. Collectively, these findings provide a foundation for future studies to elucidate the biological functions of IDD paralogs and investigate the regulatory mechanisms underlying shoot development and axillary bud outgrowth in bamboo species.

## Data Availability

The data presented in this study are available in the article, Supplementary Materials, and online repositories.

## Supplementary Materials

The following supporting information can be downloaded at: https://www.mdpi.com/article/10.3390/genes16091036/s1, Table S1: Physical and chemical properties analysis of the DlIDD genes; Table S2: Collinearity analysis results of *D. latiflorus* (intraspecific and interspecific); Table S3: Ka/Ks ratios for paralogous gene pairs; Table S4: Prediction of cis-regulatory elements in promoter regions of *DlIDDs*; Table S5: RT-qPCR primers in *D. latiflorus*; Table S6: q-PCR data.
